# miR 204-5p inhibits apoptosis in dacarbazine-treated melanoma cells

**DOI:** 10.32604/or.2022.025816

**Published:** 2022-11-10

**Authors:** NADEZHDA PALKINA, EKATERINA SERGEEVA, TATIANA RUKSHA

**Affiliations:** Krasnoyarsk State Medical University, Krasnoyarsk, 660022, Russian Federation

**Keywords:** Cell cycle, senescence, Ki-67, microRNA

## Abstract

Melanoma is one of the most aggressive types of malignant tumors, commonly affecting young individuals. The treatment of metastatic tumors remains obscure due to the resistance of tumor cells to drugs mediated by various mechanisms. The acquisition of a resistant phenotype is associated with both genetic and epigenetic alterations in cancer cells. Therefore, the current study aimed to investigate whether microRNA (miR)-204-5p could promote alterations in the cell cycle and apoptosis of dacarbazine (DTIC)-treated melanoma cells. Quantitative real time PCR showed that transfection of DTIC-treated SK-MEL-2 melanoma cells with miR-204-5p mimics significantly upregulated miR-204-5p. However, flow cytometric analysis revealed that the proportion of cells in different phases of the cell cycle remained unchanged. Additionally, the proportion of early apoptotic cells was notably enhanced following cell treatment with DTIC, accompanied by a profound increase in Ki-67 negative cells, as verified by an immunofluorescence assay. Furthermore, miR-204-5p overexpression reduced the percentage of early apoptotic DTIC-treated melanoma cells. The proportion of Ki-67 negative cells was only increased by 3%. Overall, the results of the current study indicated that miR-204-5p overexpression could mostly attenuate cell apoptosis in DTIC-treated cells rather than promote their transition from the G0 phase of the cell cycle in response to chemotherapeutic agent-induced stress.

## Introduction

Melanoma is one of the most aggressive types of malignant tumor and mostly affects young individuals [1]. The mortality rate of melanoma remains stable, but relatively high [2]. Recently, new effective approaches for treating melanoma have been developed. Therefore, BRAF-targeted therapy, based on the inhibition of the mitogen‑activated protein kinase (MAPK) pathway, has spurred a revolution in the treatment of advanced melanoma [3]. However, chemotherapy remains the standard treatment approach for disseminated melanoma and is used as a reference drug for new anti-cancer therapies in patients with melanoma. However, the mechanisms underlying low efficacy and drug resistance in cancers treated with chemotherapeutic agents remain elusive.

MicroRNAs (miRs/miRNAs) are small non-coding RNAs, 17–25 nt in length, that play a significant role in the regulation of gene expression. miRNAs can act both as oncogenes and oncosuppressor genes in cancer cells. A study revealed that miR-340-5p could inhibit colon cancer cell migration via downregulating Ras homolog family member A, a cell-specific cytoskeletal protein [4]. Additionally, the miR-let-7b family could inhibit breast cancer progression partially via regulating Aldolase C expression [5]. On the other hand, another study demonstrated that miR-30b-5p promoted breast cancer cell metastasis [6]. Furthermore, miR-96-5p promoted tumorigenic changes in gene expression and was associated with the development of ovarian cancer [7].

miR-204-5p is a member of the miRNA oncosuppessor family, consisting of miR-204-5p and miR-211 [8]. A study showed that miR-204-5р upregulation promoted cell cycle blockage in gastric cancer cells [9]. By contrast, miR-204-5р downregulation promoted the transition of hepatocellular carcinoma cells to the G1/S phase of the cell cycle and was associated with disease progression [10]. The ectopic expression of miR-204-5р could also attenuate breast cancer cell proliferation [11]. Previous studies from our laboratory revealed that inhibition of miR-204-5p could reduce the proliferation rate of melanoma cells [12]. Additionally, miR-204-5p could regulate melanoma cell proliferation via targeting forkhead box C1 transcription factor [13]. In patients exposure to chronic hypobaric hypoxia, miR-204-5p upregulation was associated with reduced melanoma specific-survival [14]. Another study demonstrated that miR-204-5р was overexpressed in A375 melanoma cells and in the vemurafenib-resistant melanoma cell clone [15]. Furthermore, Diaz-Martines et al. suggested that the treatment of A375 melanoma cells with vemurafenib upregulated miR-204-5p, thus resulting in the development of cell resistance to BRAF inhibitor. It has been also reported that both Ras and MAPK upregulation are involved in drug resistance [16].

The expression levels of miRNAs are associated with chemotherapy efficiency. Therefore, a previous study showed that the decreased expression levels of miR-31-3p were associated with improved survival rate in patients with metastatic colorectal cancer treated with the anti-EGFR antibody, cetuximab [17].

Additionally, another study demonstrated that patients sensitive to oxaliplatin/capecitabine (XELOX) chemotherapy exhibited decreased expression levels of miR-17-92 [18].

Therefore, the current study aimed to reveal how DTIC could affect the levels of miR-204-5p in melanoma cells.

## Materials and Methods

### Cell lines

The current study was approved by the Krasnoyarsk State Medical University Local Ethics Committee (protocol no. 92; issued on October 25, 2019; Krasnoyarsk, Russian Federation). All experiments were performed in SK-MEL-2 melanoma cells (ATCC® HTB-68™) which were provided by Biolot LLC (St. Petersburg, Russian Federation), cultured in RPMI-1640 medium with L-glutamine (PanEko, Moscow, Russian Federation) supplemented with 10% FBS (Gibco; Thermo Fisher Scientific Inc., New York, USA) and antibiotic/antimycotic solution (100X; Gibco; Thermo Fisher Scientific Inc., Grand Island, USA) in the MSO-5AC incubator (Sanyo Electric Co., Ltd., Osaka, Japan) with 5% CO_2_ at 37°C.

### Determination of the half-maximal (50%) inhibitory concentration (IC_50_)

The IC_50_ value for dacarbazine (DTIC) was determined using the colorimetric 3-(4,5-dimethylthiazol-2-yl)-2,5-diphenyltetrazolium bromide (MTT) method [19]. Briefly, melanoma cells at a density of 2 × 10^5^ cells/well were seeded into a 96-well plate without any intervention for 24 h. Subsequently, different concentrations (250, 500, 750 and 1000 mg/L) of a DTIC stock solution (MilliporeSigma, Saint Louis, USA) in DMSO (Panreac Quimica SA, Barcelona, Spain) was added into each well and melanoma cells were cultured for an additional 72 h without changing the medium. Following incubation, the culture medium containing DTIC was removed and 135 μl fresh culture medium supplemented with 15 μl MTT solution (Invitrogen; Thermo Fisher Scientific Inc., Eugene, USA) in PBS (concentration, 5 mg/ml; VWR International GmbH, LL AMRESCO, Solon, USA) was added into each well. Cells were treated with MTT solution for 4 h in a CO_2_-incubator. To assess the viability of cells treated with different concentrations DTIC, the accumulation intensity of the MTT-metabolite, formazan dye, was determined by measuring the optical density in each well at a wavelength of 495 and 620 nm using the Anthos 2010 ELISA spectrophotometer (Biochrom Ltd, Cambridge, England). The IC_50_ value was calculated using the Microsoft® Excel program (Microsoft Corporation, USA).

### Transient transfection of SK-MEL-2 cells with miR-204-5p-mimics

To determine the effects of miR-204-5p on DTIC-treated melanoma cells, cells were transfected with a synthetic specific analogue (mimic) for hsa-miR-204-5p (mature miRNA sequence, UUCCCUUUGUCAUCCUAUGCCU; cat. no. #4464066; MC11116; *mir*Vana® miRNA mimic; Invitrogen; Thermo Fisher Scientific Inc., Carlsbad, USA) or the corresponding negative control miRNA or corresponding positive control (*mir*Vana™ miRNA Mimic; NC #1; cat. no. #4464058; mirVanaTM miRNA Mimic miR-1 Positive Control; cat. no. #4464062, ThermoFisher Scientific, Carlsbad, USA) using Lipofectamine® 3000 reagent (both from Invitrogen; ThermoFisher Scientific, Carlsbad, USA). The transfection was carried out in FBS- and antimicrobial complex-free medium in a CO_2_-incubator at a cell density of 0.5–1 × 10^6^, according to the manufacturer’s instructions. The transfection conditions, as well as the amount of each transfection agent, were selected using the multiple titration method, followed by assessment of the transfection efficiency. Therefore, SK-MEL-2 cells were transfected with 500 nM miR-204-5p or NC mimics for 24 h.

### Cell cycle distribution (G0-phase detection)

To determine the proportion of melanoma cells into the G0 phase of the cell cycle, flow cytometric analysis was performed. Briefly, melanoma cells were removed from the surface of the culture vessel using 0.25% trypsin/EDTA solution (Gibco, Life Technologies, Paisley, UK), washed with PBS (VWR International, LL AMRESCO, Solon, USA) and fixed with 10% formaldehyde solution for 30 min at room temperature. The cell membranes were permeabilized using 0.1% Triton X-100 (GERBU Biotechnik GmbH, Heidelberg, Germany), washed with PBS, followed by incubation with antibody against Ki-67 conjugated to fluorescein (FITC; dilution, 1:100 in 10% FBS; eBioscience, Invitrogen, Carlsbad, USA) for 4 h at room temperature. The cells were then washed again with PBS and stained with propidium iodide solution (PI; 100 μg/ml; Invitrogen, Thermo Fisher Scientific, Inc., Blaiswijk, Netherlands) for 20 min. Samples containing at least 5 × 10^5^ stained cells were analyzed on a Navios flow cytometer (Beckman Coulter, Inc., Miami, USA) using a blue laser (wavelength, 488 nm) and detector filters. The passbands for FITC and PI were 530/30 and 610/20 nm, respectively. The cytometric results were processed using Navios Software v. 1.2 and Kaluza v. 2.1.1 (Beckman Coulter, Inc., Miami, USA). The gating of cells at different stages of the cell cycle was performed in a logarithmic mode. Cells in the G0 phase of the cell cycle were considered as Ki-67 negative cells and exhibited low PI levels. The above cells were gated in the range of up to 100 on the Ki-67-FITC fluorescence scale (negative) and in the range of 0.7–1.3 relative units fluorescence by PI.

### Real time PCR

To determine the gene expression levels, total RNA was isolated from melanoma cells with a set of reagents designed for the isolation of total RNA from cell cultures (diaGene; Dia-m, Moscow, Russian Federation) followed by real time PCR. To measure the expression levels of miRNAs and mRNAs, total RNA was reverse transcribed into cDNA using MMLV RT kit (Evrogen, Moscow, Russian Federation). To detect miRNA expression levels, each RT-PCR reaction consisted of 3 μl RNA, 1.5 μl 5xRT primers, provided by the corresponding miRNA kit (cat. no. #4427975; Applied Biosystems; Thermo Fisher Scientific Inc., Foster City, USA) and 1.5 μl random decanucleotide primers, provided by the MMLV RT kit. The thermocycling condition for RT-PCR was 70°C for 2 min followed by cooling on ice. Subsequently, 5.5 μl of the above reaction mixture was added to the RT-PCR reaction mixture 2, consisting of 1 μl dNTP mix, 1 μl 1,4-dithiothreitol, 2 μl 5x first standard buffer, 0.5 μl MMLV reverse transcriptase and 1 μl nuclease-free water. The reaction was carried out in a thermostat at 37°C for 50 min followed by heating for 10 min at 70°C to stop the reaction. Then, 2 μl/sample of the obtained cDNA was amplified on the StepOneTM Real-Time PCR-System (Applied Biosystems; Thermo Fisher Scientific Inc., Singapore) using the following thermocycling conditions: 50°C for 2 min, 95°C for 10 min, followed by 40 cycles at 95°C for 15 s and 60°C for 1 min. The fluorescent signal of ROX (carboxy-X-rhodamine) was also detected. The reaction mixture (total volume, 18 μl) used to determine the expression levels of both miRNAs and mRNAs consisted of 1 μl 20x primer TaqMan™ Gene Expression Assay (cat. no. #4331182; Applied Biosystems; Thermo Fisher Scientific Inc., USA) and TaqMan™ MicroRNA & Non-coding RNA Assay (cat. nos. #4427975 and 4440886; Applied Biosystems; Thermo Fisher Scientific Inc., USA), 8 μl 2.5-fold reaction mixture for RT-PCR in the presence of ROX (Syntol JSC) and 9 μl nuclease-free water. The Hs00702289_s1 (cat. no. #Hs99999903_m1) primers were used to detect the mRNA expression levels of twinfilin actin binding protein 1 (TWF1). Additionally, the corresponding primers for detecting the expression levels of miR-204-5p (cat. no. #000508) and miR-211 (cat. no. #000514) were purchased. The relative mRNA expression levels were normalized to those of ACTB (cat. no. #Hs99999903_m1) and GAPDH (cat. no. #Hs99999905_m1), while those for miRNAs were normalized to the expression levels of RNU6B (cat. no. #001093) and SNO234 (cat. no. #001234). The data were analyzed using the ∆∆Ct-method [20]. To determine the expression levels of different endogenous normalizing controls, the geometric mean of the expression levels was calculated.

### Immunofluorescence assay

Cells in the G0 phase of the cell cycle were measured using immunofluorescence. Based on a visual method, cell proliferation was assessed via detecting the presence of the intranuclear protein Ki-67. This protein is a marker of actively dividing cells and is absent from cells in the G0 phase. For immunofluorescence assays, cells were grown as a monolayer, washed with PBS and fixed with 10% formaldehyde solution for 30 min. Subsequently, the cell membranes were permeabilized using 0.5% Triton X-100 solution (GERBU Biotechnik GmbH, Heidelberg, Germany) for 10 min, followed by washing and blocking with 10% FBS solution for 1 h at room temperature. Then, the cells were first incubated with a solution of primary polyclonal rabbit anti-Ki-67 antibodies (dilution, 1:100; Abcam Inc, Cambridge, UK) at 4°C and then with a solution of secondary goat anti-rabbit antibodies (Alexa Fluor® 488 IgG (H + L); dilution, 1:200; Invitrogen, Thermo Fisher Scientific, Inc., Eugene, USA) for 1 h at room temperature. Nuclear DNA was stained with DAPI (Merck KGaA) at a concentration of 1 mg/ml for 15 min at room temperature. The stained cells were observed at a magnification of x460 using the Floid® Cell Imaging Station fluorescence module (Floid Software; version #22809; Thermo Fisher Scientific, Inc., Eugene, USA). The nuclei of living proliferating cells were stained greenish-blue, while those of living non-proliferating cells were stained blue. Cells were counted in at least 10 fields of view.

### Apoptosis assay by flow cytometry

To determine the proportion of viable, apoptotic and necrotic cells, an Annexin A5-FITC/7-AAD reagent kit (BeckmanCoulter, Inc., Marseille, France) was used, according to the manufacturer’s instructions. Briefly, cells were removed with 0.25% trypsin-EDTA solution (Gibco; Thermo Fisher Scientific Inc., Paisley, UK), washed with PBS and resuspended in 100 μl binding buffer, which was included in the reagent kit. Subsequently, melanoma cells at a density of 5 × 10^5^ were incubated with 10 μl Annexin V-FITC and 20 μl 7-AAD on ice for 15 min in the dark. Following incubation, the cells were supplemented with 400 μl binding buffer, mixed and analyzed on the Cytomics FC-500 flow cytometer (Beckman Coulter, Inc., Miami, USA) using the CXP software (version 2.2; Beckman Coulter, Inc., Miami, USA). Based on the analysis, the stained cells were divided into the following four groups: Viable cells (Annexin-V^−^ 7AAD^−^); early apoptotic cells (Annexin-V^+^ 7AAD^−^), late apoptotic/necrotic cells (Annexin-V^+^ 7AAD^+^) and necrotic cells/cell debris (Annexin-V^−^ 7AAD^+^).

### Statistical analysis

All statistical analyses were carried out using the Statistica 7.0 (StatSoft, Moscow, Russian Federation) software package. The results were analyzed using Mann-Whitney *U*-test and Fisher’s exact test. Data are expressed as the mean ± standard error of the mean (M ± m). *p* < 0.05 was considered to indicate a statistically significant difference.

## Results

**Figure 1:**
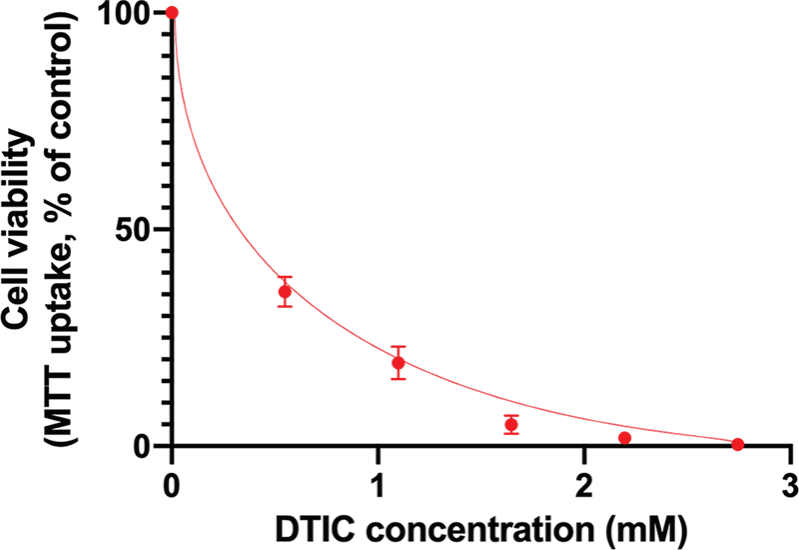
Results of the MTT-assay to determine the half-maximal inhibitory concentration of dacarbazine.

The results of the MTT assay showed that IC_50_ for SK-MEL1 cells was 0.31 mmol of DTIC (Fig. 1). However, further in our work, we used a concentration of dacarbazine equal to 4хIC_50_ in order to be able to obtain surviving cells that are resistant to this agent. Thus, the DTIC concentration was 1.2 mmol.

**Figure 2:**
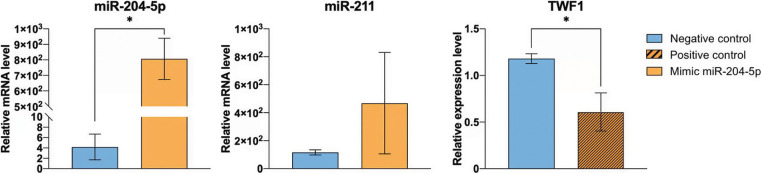
Transfection efficiency of SK-MEL-2 melanoma cells treated with 1.2 mM dacarbazine with miR-204-5p mimics. The expression levels of miR-204-5p, miR-211 and twinfilin 1 were measured using reverse transcription-quantitative PCR. *p < 0.05 (n = 3). miR-204-5p, microRNA 204-5p.

The effect of miR-204-5p overexpression following cell treatment with DTIC was investigated by real time qPCR. The expression levels of miR-204-5р were significantly increased in melanoma cells transfected with miR-204-5p mimics compared with the NC group. Additionally, the expression levels of miR-204-5p were increased by 192.81 times in miR-204-5p overexpressing DTIC-treated cells. miR-211 belongs to the same family with miR-204-5p. Therefore, to verify transfection specificity, the expression levels of miR-211 were detected in miR-204-5p overexpressing and NC cells. The results revealed that the expression of miR-211 remained unchanged, thus verifying transfection specificity. The transfection efficiency of SK-MEL-2 melanoma cells with miR-204-5p mimics was also indirectly verified via detecting the mRNA expression levels of TWF1 gene. TWF1 is validated as a direct target of the positive control miRNA, miR-1. Herein, the expression levels of TWF1 were significantly reduced by 1.94 times in SK-MEL-2 melanoma cells transfected with positive control miRNAs compared with those transfected with NC ones (Fig. 2).

**Figure 3:**
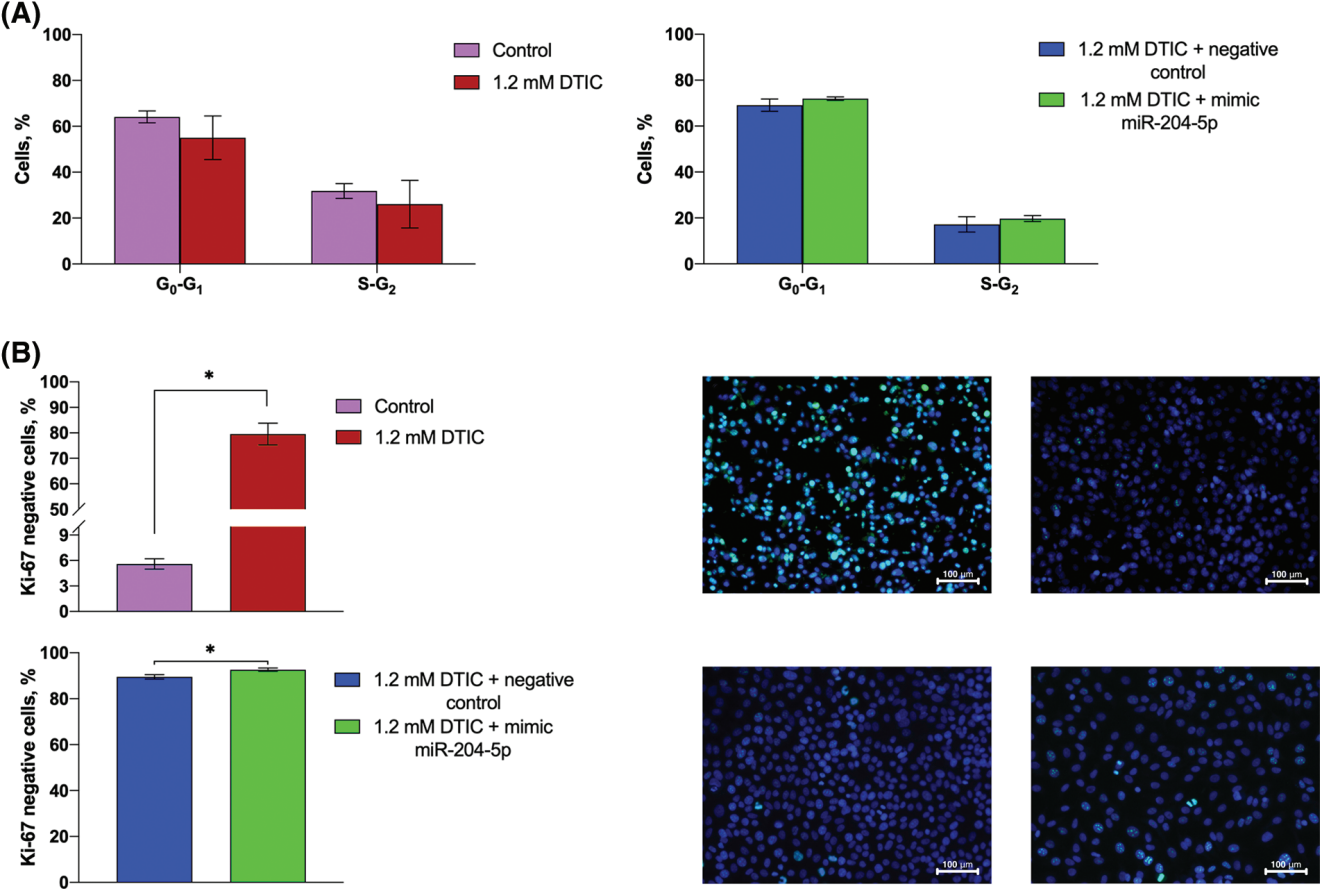
Treatment of miR-204-5p overexpressing SK-MEL-2 melanoma cells with DTIC cannot alter the proportion of cells in the G0/G1 and S/G2 phases of the cell cycle, while it elevates the proportion of Ki-67 negative cells. (A) The proportion of SK-MEL-2 melanoma cells in the G0/G1 and S/G2 phases of the cell cycle prior and after treatment with 1.2 mM DTIC, and following miR-204-5p overexpression in DTIC-treated cells were measured by flow cytometry. (B) The proportion of Ki-67 negative cells prior and after treatment with 1.2 mM DTIC and following miR-204-5p overexpression into DTIC-treated cells was determined by immunocytochemistry. In the micrographs, the nuclei of Ki-67-negative cells were stained dark-blue, while no turquoise glow was observed. *p < 0.05 (n = 3). DTIC, dacarbazine; miR-204-5p, microRNA 204-5p.

The proportion of SK-MEL-2 melanoma cells in the G0/G1 and S/G2 phases of the cell cycle remained unchanged after cell treatment with DTIC and transfection with miR-204-5p mimics (Fig. 3A). However, immunohistochemical staining to detect Ki-67 negative cells revealed that the rate of Ki-67 negative cells was enhanced by 14.21 times (5.60 *vs*. 79.55%) following cell treatment with DTIC. Furthermore, transfection of DTIC-treated melanoma cells with miR-204-5p mimics slightly elevated the percentage of Ki-67 negative cells (89.50 *vs*. 92.65%; Fig. 3B).

**Figure 4:**
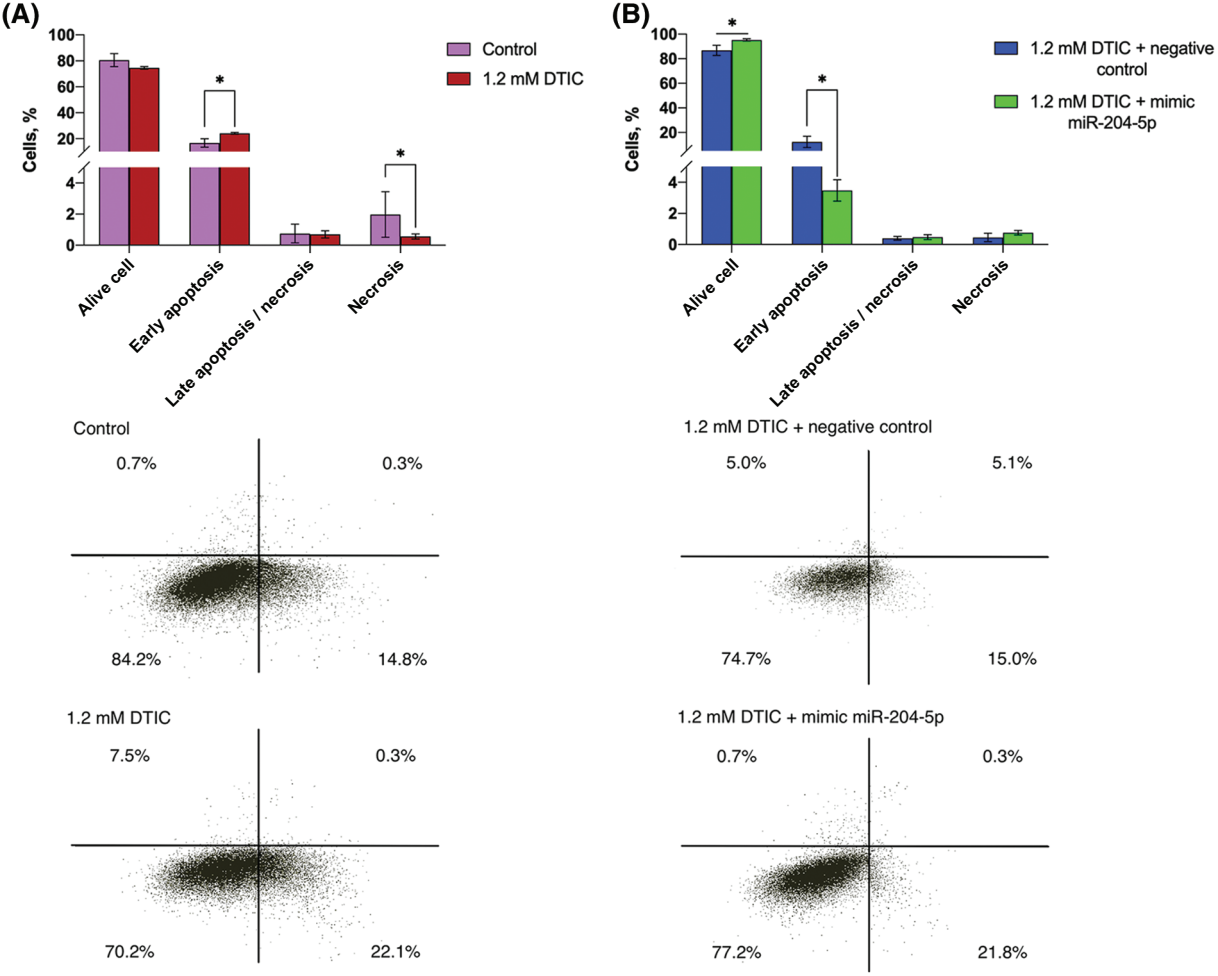
Treatment of SK-MEL-2 melanoma cells with DTIC enhances the proportion of early apoptotic cells and decreases that of necrotic cells. Treatment of miR-204-5p overexpressing SK-MEL-2 melanoma cells with 1.2 mM DTIC increases the proportion of alive cells and reduces that of early apoptotic cells. (A) The proportion of alive, apoptotic and necrotic cells after treatment with 1.2 mM DTIC was measured by flow cytometry. *p < 0.05 (n = 3). (B) The proportion of alive, apoptotic and necrotic cells after treatment with 1.2 mM DTIC and transfection with miR-204-5p mimics was measured by flow cytometry. *p < 0.05 (n = 3). DTIC, dacarbazine; miR-204-5p, microRNA 204-5p.

The early apoptotic rate was elevated by 1.45 times (16.68 *vs*. 24.12%) after cell treatment with DTIC, while the percentage of necrotic cells was reduced by 3.54 times (1.98 *vs*. 0.56%). Subsequent transfection of DTIC-treated melanoma cells with miR-204-5p mimics moderately increased the proportion of alive cells (86.78 *vs*. 95.29%) and significantly reduced the percantage of early apoptotic cells by 3.57 times compared with the NC group (12.36 *vs*. 3.47%; Fig. 4).

## Discussion

DTIC, a chemotherapy drug used for melanoma, is an alkylating agent [21]. This group of agents result in the formation of O6-alkylguanine derivatives. It has been reported that O-6-methylguanine-DNA-methyltransferase (MGMT), a DNA repair enzyme, plays a significant role in DTIC toxicity. Therefore, a study demonstrated that the methylation of MGMT promotor was associated with the treatment efficacy of DTIC [22]. However, the poor efficacy of DTIC treatment in patients with melanoma has been recently reported [23].

In the present study, the proportion of cells in various phases of the cell cycle remained unchanged after DTIC treatment and miR-204-5p overexpression. The successful transfection of SK-MEL-2 melanoma cells with miR-204-5p mimics was confirmed directly and indirectly via detecting the mRNA expression levels of TWF1. The results indicated that miR-204-5p was significantly overexpressed.

A previous study from our laboratory revealed that treatment of melanoma cells with DTIC resulted in profound alterations in the expression profile of miRNAs. Therefore, it was hypothesized that the increased expression levels of several miRNAs could be involved in the regulation of intracellular signaling and could play a crucial role in the pathogenesis of melanoma. These signaling pathways included «pathways in cancer», «phosphoinositide 3-kinase (PI3K)/AKT signaling pathway», «MAPK signaling pathway», «Ras signaling pathway» and «transcriptional misregulation in cancer» [24].

miRNAs can regulate the efficacy of antitumor drugs. The effect of antitumor therapy-mediated changes in the expression levels of miRNAs on cell autophagy, apoptosis and necrosis has been previously reported [25]. Therefore, a study demonstrated that miR-153-3p could inhibit ATG5, thus resulting in increased sensitivity of A375 and M14 melanoma cells to DTIC, and enhanced cell apoptosis and autophagy [26]. The circular RNA-mediated diminished miR-874-3p levels enhanced the sensitivity of melanoma cells to bortezomib and promoted cell apoptosis [27]. Additionally, miR-377-3p downregulation in non-small cell lung cancer cells increased their sensitivity to cisplatin and promoted cell apoptosis via disintegrin and metalloproteinase domain-containing protein [28].

The results of the present study showed that cell treatment with DTIC increased the ratio of early apoptotic cells. This effect could be triggered by several mechanisms, including the activation of ATR kinase and failed DNA repair processes [29]. By contrast, miR-204-5p overexpression in DTIC-treated cells notably reduced the proportion of early apoptotic melanoma cells. miR-204-5p could modulate cell apoptosis via several target genes. Cell apoptosis can be both triggered and inhibited by miR-204-5p. Therefore, a study revealed that miR-204-5p overexpression could trigger apoptosis via inhibiting the prooncogenic transcriptional factor early B-cell factor 2 in osteosarcoma cells [30]. In gastric cancer cells, miR-204-5p could downregulate its putative target gene, human epidermal growth factor receptor-2, thus promoting cell apoptosis [31]. On the other hand, miR-204-5p downregulation suppressed the progression of renal cell carcinoma via inhibiting cell proliferation, migration and invasion, and promoting cell apoptosis [32]. Herein, the cell apoptosis rate did not exceed 50%, which was in line with the insufficient clinical efficacy of DTIC in patients with melanoma. Additionally, the proportion of Ki-67 negative cells was notably elevated following the treatment of melanoma cells with DTIC.

Ki-67 protein is only expressed in actively proliferating cells and is used as a marker of enhanced cell proliferation in patients with malignant tumor. A previous study demonstrated that cell transfection with oligonucleotides blocking Ki-67 expression could attenuate cell proliferation, while stable Ki-67 expression levels were necessary for maintaining normal propagation rate in several cell lines [33]. Additionally, increased Ki-67 expression levels were associated with poor survival rate in patients with disseminated cutaneous melanoma [34]. In the current study, treatment of melanoma cells with DTIC not only induced cell apoptosis, but also promoted the transition of proliferative cancer cells into the G0 phase of the cell cycle.

Another study revealed that treatment of SK-MEL-19 and SK-MEL-27 melanoma cells with prodiginine induced G1/G0 phase arrest and cell apoptosis via downregulating survivin [35]. Furthermore, the BRAF^V600E^-specific inhibitor, vemurafenib, could inhibit the MAPK pathway, which in turn promoted cell cycle arrest at the G0/G1 phase of the cell cycle, eventually leading to melanoma cell apoptosis [36].

Cell cycle progression is affected by several cell cycle regulators such as cyclin-dependent kinases (CDKs), CDK inhibitors (CKI) and retinoblastoma (Rb) family proteins. Therefore, activation of the p21/27 complex could inhibit CDKs, thus preventing cell cycle initiation and progression. Another study suggested that the Rb/E2f complex could trigger global chromatin repression and NANOG expression, thus promoting cancer cell transition to the G0 phase of the cell cycle [37]. It has been also reported that several factors can affect G0 phase arrest in melanoma cells, including downregulation of melanocytic lineage-specific transcription factor, inactivation of mammalian target of rapamycin/PI3K/AKT signaling, alterations in the liver kinase B1/AMP-activated protein kinase pathway and inactivation of phosphatase and tensin homolog [38].

It has been reported that miRNAs can trigger cell cycle arrest at G0/G1phase and support G0 cell subpopulation on the post-transcriptional level [37]. Therefore, bone marrow endothelial cell-secreted miR-126 could enhance the transition of tyrosine kinase inhibitor-treated chronic myeloid leukemia cells to G0 phase via targeting sprouty-related EVH1 domain-containing protein 1, thus promoting alterations in the RAS/MAPK/extracellular signal-regulated kinase pathway [39]. In addition, a previous study suggested that miR-190 upregulation was associated with increased percentage of cells in the G0 phase of the cell cycle in patients with breast carcinoma, glioblastoma and osteosarcoma. miR-190 is involved in the regulation of several tumor suppressor genes, including CASP1, IFI6, IFITM2 and OAS1, which are associated with the induction of cell apoptosis and interferon response pathways [40]. Among the miRNAs that regulate cell cycle and, therefore, cancer cell transition to the G0 phase of the cell cycle, miR-204-5р is considered as an oncosuppressor gene.

Therefore, herein, DTIC-treated cells were transfected with miR-204-5p mimics to overexpress miR-204-5p. A study demonstrated that miR-204-5p was involved in the regulation of cell proliferation [12]. Therefore, the effect of miR-204-5p overexpression on cell cycle distribution and the percentage of Ki-67 cells in DTIC-treated melanoma cells was investigated. A moderate increase in the percentage of Ki-67 negative cells was only observed, possibly due to the transition of melanoma cells to the G0 phase.

Overall, the results of the current study demonstrated that miR-204-5p could affect cancer cell resistance to chemotherapeutic drugs mostly via modulating apoptosis. Therefore, miR-204-5p could be involved in the apoptosis of melanoma cells triggered by DTIC or miR-204-5p overexpression rather in their transition to the G0 phase of the cell cycle.

The results from these studies suggest that miR-204-5p levels modulation can be considered as a valid therapeutic approach for combination treatment of melanoma.

## Data Availability

The data and materials used in the study are available on request.
